# *catena*-Poly[[aqua­(1,10-phenanthroline)cobalt(II)]-μ-4,4′-(propane-1,3-diyldi­oxy)dibenzoato]

**DOI:** 10.1107/S1600536809035089

**Published:** 2009-09-05

**Authors:** Su-Mei Shen

**Affiliations:** a Department of Applied Engineering, Zhejiang Economic and Trade Polytechnic, 310018 Hangzhou, People’s Republic of China

## Abstract

In the title compound, [Co(C_17_H_14_O_6_)(C_12_H_8_N_2_)(H_2_O)]_*n*_, the Co^II^ atom is coordinated by a monodentate 4,4′-(propane-1,3-diyldi­oxy)dibenzoate (cpp) dianion, a water mol­ecule and a chelating 1,10-phenanthroline (phen) ligand. A symmetry-generated cpp ligand completes the CoN_2_O_3_ trigonal-bipyramidal geometry for the metal ion, with the N atoms occupying both equatorial and axial sites. The bridging cpp ligands form chains propagating in [110] and O—H⋯O hydrogen bonds consolidate the packing.

## Related literature

For a related structure, see: Chen & Liu (2002 ▶). For background to metal-organic frameworks, see: Kitagawa *et al.* (2004 ▶); Liu *et al.* (2009 ▶); Schokecht & Kempe (2004 ▶).
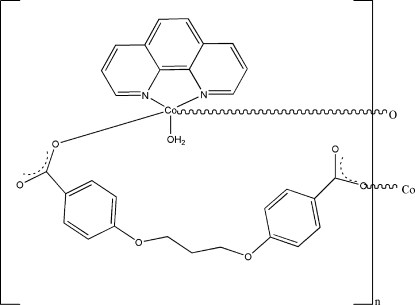


## Experimental

### 

#### Crystal data

[Co(C_17_H_14_O_6_)(C_12_H_8_N_2_)(H_2_O)]
*M* *_r_* = 571.43
Triclinic, 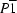

*a* = 8.5967 (17) Å
*b* = 11.432 (2) Å
*c* = 14.423 (3) Å
α = 68.433 (3)°
β = 87.673 (4)°
γ = 74.635 (4)°
*V* = 1268.5 (4) Å^3^
*Z* = 2
Mo *K*α radiation
μ = 0.73 mm^−1^
*T* = 298 K
0.23 × 0.14 × 0.11 mm

#### Data collection

Bruker APEXII area-detector diffractometer
Absorption correction: multi-scan (*SADABS*; Bruker, 2005 ▶) *T*_min_ = 0.850, *T*_max_ = 0.924
6503 measured reflections
4499 independent reflections
2242 reflections with *I* > 2σ(*I*)
*R*_int_ = 0.032

#### Refinement

*R*[*F* ^2^ > 2σ(*F* ^2^)] = 0.039
*wR*(*F* ^2^) = 0.063
*S* = 0.91
4499 reflections
358 parameters
3 restraints
H atoms treated by a mixture of independent and constrained refinement
Δρ_max_ = 0.19 e Å^−3^
Δρ_min_ = −0.21 e Å^−3^

### 

Data collection: *APEX2* (Bruker, 2005 ▶); cell refinement: *SAINT* (Bruker, 2005 ▶); data reduction: *SAINT*; program(s) used to solve structure: *SHELXS97* (Sheldrick, 2008 ▶); program(s) used to refine structure: *SHELXL97* (Sheldrick, 2008 ▶); molecular graphics: *ORTEPIII* (Burnett & Johnson, 1996 ▶), *ORTEP-3 for Windows* (Farrugia, 1997 ▶) and *PLATON* (Spek, 2009 ▶); software used to prepare material for publication: *SHELXL97*.

## Supplementary Material

Crystal structure: contains datablocks I, global. DOI: 10.1107/S1600536809035089/hb5085sup1.cif

Structure factors: contains datablocks I. DOI: 10.1107/S1600536809035089/hb5085Isup2.hkl

Additional supplementary materials:  crystallographic information; 3D view; checkCIF report

## Figures and Tables

**Table 1:** Hydrogen-bond geometry (Å, °)

*D*—H⋯*A*	*D*—H	H⋯*A*	*D*⋯*A*	*D*—H⋯*A*
O1*W*—H1*WB*⋯O2^i^	0.885 (15)	1.847 (16)	2.729 (3)	175 (3)
O1*W*—H1*WA*⋯O5^ii^	0.885 (17)	1.804 (17)	2.657 (3)	161 (3)

Symmetry codes: (i) 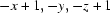; (ii) 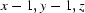.

**Fig. 1.**
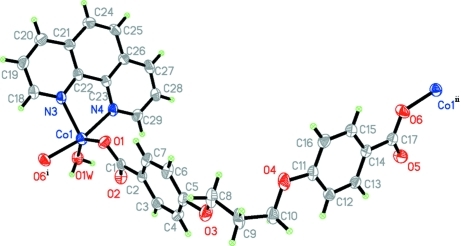
View of a fragment of (I). Ellipsoids are drawn at the the 30% probability level. H atomsare shown as spheres of arbitrary radii. [symmetry codes: (i) x-1, y-1, z; (ii) 1+x, y+1, 3+z]

**Fig. 2.**
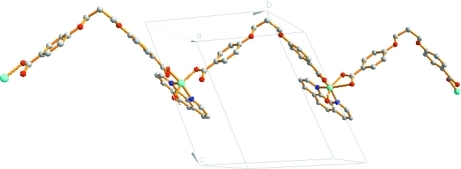
Partial packing of (I) showing the formation of a chain along c axis.

## supplementary crystallographic information

### Comment

Design of effective ligands and the proper choice of metal centers are the keys to design and construct novel metal-organic frameworks (Kitagawa *et al.*,  2004; Schokecht & Kempe, 2004). Polycarboxylate ligands have received considerable attention, owing to the variety of their coordination modes and structural features. 4,4'-(propane-1,3-diyldioxy)dibenzoic acid (H_2_cpp) is a potential multi-dentate ligand with a versatile coordination mode, which has been used in self-asssembled porous coordination synthesis (Liu *et al.*,  2009).

The title compound, (I), was constructed by two kinds of bridging and chelating ligands under mild condition, H_2_cpp and phen which were self-assembled to a one-dimensional neutral metal-organic compound. In this paper, the crystal structure of (I) is presented.

As illustrated in Fig. 1, Co^II^ adopts a trigonal bispyramidal geometry, generated by three O atoms from two adjacent monodenated-chelating carboxylate groups and one coordinated water molecule, and two N atoms from one chelating phen ligand. The three atoms (O1, O1W and N3) in the basical plane around the Co atom, while the other two atoms (O6 and N4) locate at apical positions. The twist angle of two rings of cpp ligand is 96.8 (5)°.

The neighboring Co atoms are linked by cp ligands forming a one-dimensional chain running along a axis (Fig. 2). These chains are decorated with phen ligands alternating on two sides, which is similar with some complexes (Chen & Liu, 2002) There are no π-π interactions between rings of phen ligands due to its transplacement arrangement.

In the crystal structure, strong intermolecular O-H···O hydrogen bonds (Table 1) link the molecules into a 2D network, in which they may be effective in the stabilization of the structure.

### Experimental

The followinf quantities were mixed: (23 mg, 0.1 mmol)) Co(NO_3_)_2_ of water solution (5 ml) and H_2_CP (26 mg, 0.1 mmol), phen (0.19 mg, 0.1 mmol) and NaOH (3.8 mg, 0.09 mmol), CH_3_CN (5 ml) and heated to at 428 K for 60 h in a pressurized reactor. Slow evaporation of this solution resulted in the formation of some pink blocks of (I).

### Refinement

All H atoms attached to C atoms were fixed geometrically and treated as riding with C—H = 0.93 Å (aromatic) with U_iso_(H) = 1.2U_eq_(C). H atoms of water molecule were located in difference Fourier maps and included in the subsequent refinement using  restraints (O-H = 0.84 (2)Å and H···H = 1.38 (2)Å) with U_iso_(H) = 1.5U_eq_(O). The highest residual difference electron-density peak is 1.50Å from N3.

### Crystal data

**Table:**

[Co(C_17_H_14_O_6_)(C_12_H_8_N_2_)(H_2_O)]	*Z* = 2
*M**_r_* = 571.43	*F*(000) = 590
Triclinic, *P*1	*D*_x_ = 1.496 Mg m^−^^3^
Hall symbol: -P 1	Mo *K*α radiation, λ = 0.71073 Å
*a* = 8.5967 (17) Å	Cell parameters from 4499 reflections
*b* = 11.432 (2) Å	θ = 1.5–25.2°
*c* = 14.423 (3) Å	µ = 0.73 mm^−^^1^
α = 68.433 (3)°	*T* = 298 K
β = 87.673 (4)°	Block, pink
γ = 74.635 (4)°	0.23 × 0.14 × 0.11 mm
*V* = 1268.5 (4) Å^3^	

### Data collection

**Table:**

Bruker APEXII area-detector diffractometer	4499 independent reflections
Radiation source: fine-focus sealed tube	2242 reflections with *I* > 2σ(*I*)
graphite	*R*_int_ = 0.032
φ and ω scans	θ_max_ = 25.2°, θ_min_ = 1.5°
Absorption correction: multi-scan (*SADABS*; Bruker, 2005)	*h* = −10→8
*T*_min_ = 0.850, *T*_max_ = 0.924	*k* = −11→13
6503 measured reflections	*l* = −17→17

### Refinement

**Table:**

Refinement on *F*^2^	Primary atom site location: structure-invariant direct methods
Least-squares matrix: full	Secondary atom site location: difference Fourier map
*R*[*F*^2^ > 2σ(*F*^2^)] = 0.039	Hydrogen site location: inferred from neighbouring sites
*wR*(*F*^2^) = 0.063	H atoms treated by a mixture of independent and constrained refinement
*S* = 0.91	*w* = 1/[σ^2^(*F*_o_^2^) + (0.01*P*)^2^ + 0.001*P*] where *P* = (*F*_o_^2^ + 2*F*_c_^2^)/3
4499 reflections	(Δ/σ)_max_ < 0.001
358 parameters	Δρ_max_ = 0.19 e Å^−^^3^
3 restraints	Δρ_min_ = −0.21 e Å^−^^3^

### Special details

**Table:**

Geometry. All esds (except the esd in the dihedral angle between two l.s. planes) are estimated using the full covariance matrix. The cell esds are taken into account individually in the estimation of esds in distances, angles and torsion angles; correlations between esds in cell parameters are only used when they are defined by crystal symmetry. An approximate (isotropic) treatment of cell esds is used for estimating esds involving l.s. planes.
Refinement. Refinement of F^2^ against ALL reflections. The weighted R-factor wR and goodness of fit S are based on F^2^, conventional R-factors R are based on F, with F set to zero for negative F^2^. The threshold expression of F^2^ > 2sigma(F^2^) is used only for calculating R-factors(gt) etc. and is not relevant to the choice of reflections for refinement. R-factors based on F^2^ are statistically about twice as large as those based on F, and R- factors based on ALL data will be even larger.

### Fractional atomic coordinates and isotropic or equivalent isotropic displacement parameters (Å^2^)

**Table:**

	*x*	*y*	*z*	*U*_iso_*/*U*_eq_	
Co1	0.63053 (6)	−0.14519 (4)	0.37254 (3)	0.04796 (15)	
O1	0.6918 (3)	0.01199 (17)	0.26875 (13)	0.0552 (6)	
O2	0.5285 (3)	0.08365 (17)	0.36879 (14)	0.0568 (6)	
O6	1.4634 (3)	0.84914 (19)	0.28126 (14)	0.0557 (6)	
O5	1.2435 (3)	0.91935 (18)	0.35373 (14)	0.0599 (6)	
O3	0.6256 (3)	0.62108 (18)	0.06262 (15)	0.0663 (7)	
O4	0.9367 (3)	0.79789 (19)	0.01759 (15)	0.0645 (7)	
O1W	0.4880 (3)	−0.19495 (18)	0.49172 (15)	0.0547 (6)	
N3	0.8001 (3)	−0.2965 (2)	0.34395 (17)	0.0459 (7)	
N4	0.8312 (3)	−0.1855 (2)	0.47342 (16)	0.0479 (7)	
C5	0.6272 (4)	0.4931 (3)	0.1143 (2)	0.0478 (9)	
C15	1.2806 (4)	0.8073 (2)	0.14796 (19)	0.0446 (8)	
H15	1.3926	0.7800	0.1481	0.053*	
C2	0.6156 (4)	0.2400 (3)	0.23088 (19)	0.0372 (8)	
C17	1.3121 (5)	0.8825 (3)	0.2871 (2)	0.0442 (9)	
C14	1.2101 (4)	0.8686 (2)	0.21218 (19)	0.0388 (8)	
C11	1.0202 (5)	0.8281 (3)	0.0810 (2)	0.0466 (9)	
C22	0.9490 (4)	−0.3320 (3)	0.3897 (2)	0.0431 (8)	
C1	0.6105 (4)	0.1044 (3)	0.2937 (2)	0.0449 (9)	
C16	1.1861 (5)	0.7864 (3)	0.0841 (2)	0.0513 (9)	
H16	1.2349	0.7435	0.0424	0.062*	
C13	1.0445 (4)	0.9135 (2)	0.2059 (2)	0.0428 (8)	
H13	0.9959	0.9574	0.2469	0.051*	
C23	0.9660 (4)	−0.2726 (3)	0.4596 (2)	0.0427 (8)	
C20	1.0562 (5)	−0.4759 (3)	0.3037 (2)	0.0574 (10)	
H20	1.1410	−0.5348	0.2888	0.069*	
C12	0.9468 (4)	0.8959 (2)	0.1406 (2)	0.0462 (8)	
H12	0.8350	0.9287	0.1370	0.055*	
C7	0.7238 (4)	0.2625 (3)	0.1578 (2)	0.0491 (9)	
H7	0.7939	0.1918	0.1473	0.059*	
C18	0.7807 (4)	−0.3527 (3)	0.2806 (2)	0.0516 (9)	
H18	0.6787	−0.3314	0.2498	0.062*	
C4	0.5183 (4)	0.4717 (3)	0.1876 (2)	0.0517 (9)	
H4	0.4480	0.5422	0.1983	0.062*	
C6	0.7314 (4)	0.3883 (3)	0.0993 (2)	0.0536 (10)	
H6	0.8061	0.4013	0.0506	0.064*	
C3	0.5132 (4)	0.3464 (3)	0.2450 (2)	0.0473 (9)	
H3	0.4393	0.3334	0.2941	0.057*	
C21	1.0827 (4)	−0.4225 (3)	0.3726 (2)	0.0454 (8)	
C29	0.8459 (4)	−0.1330 (3)	0.5391 (2)	0.0566 (10)	
H29	0.7556	−0.0737	0.5489	0.068*	
C26	1.1150 (4)	−0.3060 (3)	0.5110 (2)	0.0494 (9)	
C24	1.2331 (4)	−0.4527 (3)	0.4262 (2)	0.0606 (10)	
H24	1.3225	−0.5120	0.4154	0.073*	
C9	0.7016 (4)	0.7961 (3)	−0.0633 (2)	0.0729 (12)	
H9A	0.5869	0.8383	−0.0787	0.088*	
H9B	0.7551	0.8191	−0.1255	0.088*	
C19	0.9069 (5)	−0.4420 (3)	0.2586 (2)	0.0596 (10)	
H19	0.8888	−0.4781	0.2132	0.072*	
C8	0.7272 (4)	0.6493 (3)	−0.0194 (2)	0.0680 (11)	
H8A	0.6989	0.6191	−0.0695	0.082*	
H8B	0.8396	0.6056	0.0035	0.082*	
C27	1.1243 (4)	−0.2470 (3)	0.5802 (2)	0.0615 (10)	
H27	1.2213	−0.2661	0.6159	0.074*	
C28	0.9895 (5)	−0.1616 (3)	0.5944 (2)	0.0635 (10)	
H28	0.9935	−0.1229	0.6405	0.076*	
C25	1.2484 (4)	−0.3972 (3)	0.4922 (2)	0.0581 (9)	
H25	1.3481	−0.4189	0.5259	0.070*	
C10	0.7656 (5)	0.8476 (3)	0.0062 (2)	0.0634 (10)	
H10A	0.7359	0.9423	−0.0217	0.076*	
H10B	0.7196	0.8195	0.0706	0.076*	
H1WA	0.399 (3)	−0.145 (3)	0.453 (2)	0.095*	
H1WB	0.482 (4)	−0.163 (3)	0.5397 (16)	0.095*	

### Atomic displacement parameters (Å^2^)

**Table:**

	*U*^11^	*U*^22^	*U*^33^	*U*^12^	*U*^13^	*U*^23^
Co1	0.0490 (4)	0.0497 (3)	0.0462 (3)	−0.0154 (2)	−0.0012 (2)	−0.0169 (2)
O1	0.0598 (18)	0.0395 (13)	0.0671 (15)	−0.0094 (11)	0.0061 (12)	−0.0235 (11)
O2	0.072 (2)	0.0588 (14)	0.0454 (14)	−0.0295 (12)	0.0080 (12)	−0.0181 (11)
O6	0.0438 (18)	0.0788 (16)	0.0523 (14)	−0.0163 (13)	−0.0046 (13)	−0.0324 (12)
O5	0.0539 (18)	0.0800 (16)	0.0604 (15)	−0.0118 (12)	−0.0031 (12)	−0.0462 (13)
O3	0.085 (2)	0.0444 (14)	0.0696 (16)	−0.0252 (13)	−0.0050 (14)	−0.0151 (12)
O4	0.079 (2)	0.0670 (15)	0.0612 (15)	−0.0276 (15)	−0.0163 (14)	−0.0321 (12)
O1W	0.064 (2)	0.0617 (15)	0.0437 (14)	−0.0218 (12)	0.0013 (11)	−0.0222 (12)
N3	0.053 (2)	0.0458 (16)	0.0438 (16)	−0.0165 (14)	−0.0043 (15)	−0.0190 (13)
N4	0.057 (2)	0.0510 (16)	0.0426 (16)	−0.0185 (15)	−0.0054 (14)	−0.0218 (13)
C5	0.062 (3)	0.036 (2)	0.047 (2)	−0.0167 (19)	−0.0113 (19)	−0.0136 (17)
C15	0.048 (3)	0.0424 (18)	0.0453 (19)	−0.0145 (16)	0.0018 (18)	−0.0170 (16)
C2	0.044 (2)	0.0402 (19)	0.0331 (18)	−0.0136 (17)	−0.0008 (16)	−0.0176 (15)
C17	0.053 (3)	0.0387 (19)	0.043 (2)	−0.0141 (18)	0.000 (2)	−0.0160 (16)
C14	0.051 (3)	0.0331 (17)	0.0341 (18)	−0.0136 (17)	0.0003 (17)	−0.0125 (14)
C11	0.063 (3)	0.0393 (19)	0.042 (2)	−0.0215 (19)	−0.0062 (19)	−0.0131 (16)
C22	0.049 (3)	0.0407 (19)	0.040 (2)	−0.0205 (18)	0.0011 (18)	−0.0100 (16)
C1	0.048 (3)	0.045 (2)	0.048 (2)	−0.0173 (18)	−0.0128 (18)	−0.0192 (18)
C16	0.061 (3)	0.049 (2)	0.052 (2)	−0.012 (2)	0.001 (2)	−0.0296 (17)
C13	0.049 (3)	0.0384 (18)	0.045 (2)	−0.0107 (17)	0.0005 (18)	−0.0202 (15)
C23	0.045 (3)	0.046 (2)	0.038 (2)	−0.0179 (18)	−0.0025 (18)	−0.0128 (16)
C20	0.060 (3)	0.052 (2)	0.058 (2)	−0.008 (2)	0.001 (2)	−0.0235 (18)
C12	0.047 (3)	0.0418 (19)	0.049 (2)	−0.0120 (16)	−0.0071 (18)	−0.0146 (16)
C7	0.067 (3)	0.043 (2)	0.047 (2)	−0.0169 (18)	0.0029 (19)	−0.0259 (17)
C18	0.063 (3)	0.051 (2)	0.046 (2)	−0.0232 (19)	−0.0024 (18)	−0.0167 (17)
C4	0.049 (3)	0.040 (2)	0.067 (2)	−0.0037 (17)	−0.0044 (19)	−0.0248 (18)
C6	0.080 (3)	0.049 (2)	0.043 (2)	−0.030 (2)	0.0109 (19)	−0.0209 (17)
C3	0.047 (3)	0.048 (2)	0.047 (2)	−0.0140 (18)	−0.0013 (17)	−0.0169 (17)
C21	0.045 (3)	0.044 (2)	0.048 (2)	−0.0119 (18)	0.0055 (19)	−0.0169 (16)
C29	0.062 (3)	0.056 (2)	0.059 (2)	−0.0123 (19)	−0.003 (2)	−0.0311 (18)
C26	0.044 (3)	0.048 (2)	0.056 (2)	−0.0140 (18)	−0.0012 (19)	−0.0179 (17)
C24	0.048 (3)	0.051 (2)	0.078 (3)	−0.0065 (18)	0.001 (2)	−0.0237 (19)
C9	0.110 (4)	0.060 (2)	0.051 (2)	−0.047 (2)	−0.029 (2)	−0.0032 (18)
C19	0.071 (3)	0.060 (2)	0.056 (2)	−0.016 (2)	−0.001 (2)	−0.0324 (19)
C8	0.107 (4)	0.056 (2)	0.051 (2)	−0.044 (2)	−0.010 (2)	−0.0138 (18)
C27	0.062 (3)	0.071 (2)	0.059 (2)	−0.025 (2)	−0.015 (2)	−0.024 (2)
C28	0.061 (3)	0.071 (3)	0.070 (3)	−0.016 (2)	−0.015 (2)	−0.040 (2)
C25	0.042 (3)	0.062 (2)	0.071 (2)	−0.0132 (19)	−0.0098 (19)	−0.024 (2)
C10	0.082 (4)	0.050 (2)	0.055 (2)	−0.026 (2)	−0.023 (2)	−0.0072 (17)

### Geometric parameters (Å, °)

**Table:**

Co1—O6^i^	2.017 (2)	C13—C12	1.387 (4)
Co1—O1	2.0511 (17)	C13—H13	0.9300
Co1—O1W	2.063 (2)	C23—C26	1.395 (4)
Co1—N3	2.104 (2)	C20—C19	1.357 (4)
Co1—N4	2.143 (2)	C20—C21	1.396 (4)
O1—C1	1.265 (3)	C20—H20	0.9300
O2—C1	1.251 (3)	C12—H12	0.9300
O6—C17	1.263 (3)	C7—C6	1.392 (3)
O6—Co1^ii^	2.017 (2)	C7—H7	0.9300
O5—C17	1.253 (3)	C18—C19	1.394 (4)
O3—C5	1.373 (3)	C18—H18	0.9300
O3—C8	1.432 (3)	C4—C3	1.377 (3)
O4—C11	1.374 (3)	C4—H4	0.9300
O4—C10	1.421 (4)	C6—H6	0.9300
O1W—H1WA	0.885 (17)	C3—H3	0.9300
O1W—H1WB	0.885 (15)	C21—C24	1.427 (4)
N3—C18	1.332 (3)	C29—C28	1.393 (4)
N3—C22	1.354 (4)	C29—H29	0.9300
N4—C29	1.319 (3)	C26—C27	1.409 (3)
N4—C23	1.374 (3)	C26—C25	1.423 (4)
C5—C6	1.375 (4)	C24—C25	1.352 (3)
C5—C4	1.381 (3)	C24—H24	0.9300
C15—C16	1.376 (4)	C9—C8	1.517 (3)
C15—C14	1.387 (3)	C9—C10	1.518 (3)
C15—H15	0.9300	C9—H9A	0.9700
C2—C7	1.374 (3)	C9—H9B	0.9700
C2—C3	1.376 (3)	C19—H19	0.9300
C2—C1	1.493 (3)	C8—H8A	0.9700
C17—C14	1.496 (4)	C8—H8B	0.9700
C14—C13	1.374 (4)	C27—C28	1.366 (4)
C11—C16	1.375 (4)	C27—H27	0.9300
C11—C12	1.388 (3)	C28—H28	0.9300
C22—C21	1.409 (4)	C25—H25	0.9300
C22—C23	1.439 (3)	C10—H10A	0.9700
C16—H16	0.9300	C10—H10B	0.9700
			
O6^i^—Co1—O1	95.13 (8)	C13—C12—H12	120.9
O6^i^—Co1—O1W	90.40 (9)	C11—C12—H12	120.9
O1—Co1—O1W	142.18 (7)	C2—C7—C6	121.8 (3)
O6^i^—Co1—N3	90.17 (10)	C2—C7—H7	119.1
O1—Co1—N3	99.18 (8)	C6—C7—H7	119.1
O1W—Co1—N3	118.22 (8)	N3—C18—C19	122.8 (3)
O6^i^—Co1—N4	166.69 (9)	N3—C18—H18	118.6
O1—Co1—N4	92.61 (8)	C19—C18—H18	118.6
O1W—Co1—N4	90.10 (9)	C3—C4—C5	120.3 (3)
N3—Co1—N4	77.90 (10)	C3—C4—H4	119.8
C1—O1—Co1	101.40 (18)	C5—C4—H4	119.8
C17—O6—Co1^ii^	125.76 (19)	C5—C6—C7	119.2 (3)
C5—O3—C8	118.2 (2)	C5—C6—H6	120.4
C11—O4—C10	118.3 (2)	C7—C6—H6	120.4
Co1—O1W—H1WA	91 (2)	C2—C3—C4	121.2 (3)
Co1—O1W—H1WB	122.6 (19)	C2—C3—H3	119.4
H1WA—O1W—H1WB	103 (2)	C4—C3—H3	119.4
C18—N3—C22	117.3 (3)	C20—C21—C22	116.5 (3)
C18—N3—Co1	127.3 (2)	C20—C21—C24	125.1 (3)
C22—N3—Co1	115.16 (19)	C22—C21—C24	118.3 (3)
C29—N4—C23	117.4 (3)	N4—C29—C28	123.5 (3)
C29—N4—Co1	129.9 (2)	N4—C29—H29	118.3
C23—N4—Co1	112.55 (19)	C28—C29—H29	118.3
O3—C5—C6	124.4 (3)	C23—C26—C27	117.3 (3)
O3—C5—C4	116.0 (3)	C23—C26—C25	119.3 (3)
C6—C5—C4	119.5 (3)	C27—C26—C25	123.4 (4)
C16—C15—C14	120.5 (3)	C25—C24—C21	121.4 (3)
C16—C15—H15	119.8	C25—C24—H24	119.3
C14—C15—H15	119.8	C21—C24—H24	119.3
C7—C2—C3	118.0 (3)	C8—C9—C10	113.0 (2)
C7—C2—C1	121.0 (3)	C8—C9—H9A	109.0
C3—C2—C1	121.0 (3)	C10—C9—H9A	109.0
O5—C17—O6	124.6 (3)	C8—C9—H9B	109.0
O5—C17—C14	118.5 (3)	C10—C9—H9B	109.0
O6—C17—C14	116.8 (3)	H9A—C9—H9B	107.8
C13—C14—C15	117.9 (3)	C20—C19—C18	119.7 (3)
C13—C14—C17	121.5 (3)	C20—C19—H19	120.2
C15—C14—C17	120.6 (3)	C18—C19—H19	120.2
O4—C11—C16	116.4 (3)	O3—C8—C9	107.4 (3)
O4—C11—C12	123.8 (3)	O3—C8—H8A	110.2
C16—C11—C12	119.8 (3)	C9—C8—H8A	110.2
N3—C22—C21	123.7 (3)	O3—C8—H8B	110.2
N3—C22—C23	116.3 (3)	C9—C8—H8B	110.2
C21—C22—C23	120.1 (3)	H8A—C8—H8B	108.5
O2—C1—O1	121.5 (3)	C28—C27—C26	119.4 (3)
O2—C1—C2	120.5 (3)	C28—C27—H27	120.3
O1—C1—C2	118.0 (3)	C26—C27—H27	120.3
C11—C16—C15	120.9 (3)	C27—C28—C29	119.3 (3)
C11—C16—H16	119.6	C27—C28—H28	120.3
C15—C16—H16	119.6	C29—C28—H28	120.3
C14—C13—C12	122.6 (3)	C24—C25—C26	121.3 (3)
C14—C13—H13	118.7	C24—C25—H25	119.4
C12—C13—H13	118.7	C26—C25—H25	119.4
N4—C23—C26	123.0 (3)	O4—C10—C9	108.3 (3)
N4—C23—C22	117.4 (3)	O4—C10—H10A	110.0
C26—C23—C22	119.6 (3)	C9—C10—H10A	110.0
C19—C20—C21	120.1 (3)	O4—C10—H10B	110.0
C19—C20—H20	120.0	C9—C10—H10B	110.0
C21—C20—H20	120.0	H10A—C10—H10B	108.4
C13—C12—C11	118.2 (3)		

Symmetry codes: (i) *x*−1, *y*−1, *z*; (ii) *x*+1, *y*+1, *z*.

### Hydrogen-bond geometry (Å, °)

**Table:**

*D*—H···*A*	*D*—H	H···*A*	*D*···*A*	*D*—H···*A*
O1W—H1WB···O2^iii^	0.89 (2)	1.85 (2)	2.729 (3)	175 (3)
O1W—H1WA···O5^i^	0.89 (2)	1.80 (2)	2.657 (3)	161 (3)

Symmetry codes: (iii) −*x*+1, −*y*, −*z*+1; (i) *x*−1, *y*−1, *z*.
